# Adenosine and Kynurenic Acid Interactions: Possible Relevance for Schizophrenia Treatment?

**DOI:** 10.3389/fphar.2021.654426

**Published:** 2021-04-14

**Authors:** Sarah Beggiato, Mariachiara Zuccarini, Tommaso Cassano, Dasiel Oscar Borroto-Escuela, Patrizia Di Iorio, Robert Schwarcz, Kjell Fuxe, Luca Ferraro

**Affiliations:** ^1^ Department of Medical, Oral and Biotechnological Sciences, University of Chieti-Pescara, Chieti, Italy; ^2^ Department of Medical and Surgical Sciences, University of Foggia, Foggia, Italy; ^3^ Department of Neuroscience, Karolinska Institutet, Stockholm, Sweden; ^4^ Department of Psychiatry, Maryland Psychiatric Research Center, University of Maryland School of Medicine, Baltimore, MD, United States; ^5^ Department of Life Sciences and Biotechnology and LTTA Center, University of Ferrara, Ferrara, Italy

**Keywords:** adenosine A_1_ and A_2A_ receptors, cognition, combinatory therapy, kynurenine pathway, receptor heteromers

## Introduction

Schizophrenia is a severe and chronic mental disorder, mainly characterized by the presence of the so-called “positive” (delusions, hallucinations, disorganized thinking) and “negative” (anhedonia, blunted affect, social withdrawal) symptoms, as well as cognitive dysfunctions. Although several interrelated causes have been associated with the development of the pathology, it is generally accepted that the hyperfunction of dopaminergic and/or hypofunction of glutamatergic transmission (i.e., the so-called “combined glutamate/dopamine hypothesis of schizophrenia”) might underlie the symptoms of schizophrenia (Howes et al., 2015; Snyder and Gao, 2020). Clinical indications demonstrate that positive symptoms respond well to conventional antipsychotic medications, which mainly act as dopamine D_2_ receptor (D_2_R) antagonists, while negative symptoms and cognitive impairments are more difficult to be counteracted. Several non-D_2_R related mechanisms of action of antipsychotic drugs have been proposed over the last decades, but none has conclusively been proven effective. Furthermore, while the newer antipsychotic drugs produce fewer motor side effects than conventional “first generation” drugs, safety and tolerability concerns about weight gain and endocrinopathies often limit their use (Li et al., 2016). Thus, there is an urgent necessity for more effective and better-tolerated antipsychotic drugs, as well as to identify new molecular targets and develop mechanistically novel compounds that can address the various symptom dimensions of schizophrenia. Due to the complexity of the pathology, it seems likely, however, that a multi-target strategy, i.e., the use of multifunctional drugs or a combination of drugs affecting distinct targets, will lead to more effective therapeutic approaches.

Based on this background and recent findings, the present opinion paper was conceived to critically review possible interactions between adenosine and kynurenic acid (KYNA) in this context. These two neuromodulators may be pathophysiologically associated with schizophrenia, and a deeper understanding of their interactions may lead to the development of innovative strategies for the treatment of schizophrenia.

### Adenosine and Schizophrenia

It is well recognized that, beside dopamine and glutamate systems, the purinergic system may be also involved in the pathophysiology of schizophrenia (Lara and Souza, 2000; Krügel, 2016; Cheffer et al., 2018). In fact, the so-called “adenosine hypothesis of schizophrenia” (Lara et al., 2006; Boison et al., 2012; Hirota and Kishi, 2013; Rial et al., 2014) postulates that a reduced adenosine tone is involved in the dysregulation of glutamatergic and dopaminergic activity in schizophrenia patients. Accordingly, based on informative studies in experimental animals, adenosine receptor agonists may act as atypical antipsychotic drugs (Krügel, 2016).

Adenosine A_2A_ receptors (A_2A_Rs), which are highly expressed in the striatum and the olfactory tubercle, exert fine regulation of individual synapses (Hines and Haydon, 2014; Krügel, 2016), and their activation facilitates glutamate release and potentiates N-methyl-D-aspartate (NMDA) receptor function. As a consequence, A_2A_Rs regulate synaptic plasticity by promoting adequate (or aberrant) adaptive responses in neuronal circuits (Azdad et al., 2009; Boison and Aronica, 2015; Krügel, 2016). In general, adenosine and A_2A_R agonists induce behavioral effects similar to those of dopamine receptor (DR) antagonists used as antipsychotics (Rimondini et al., 1997; Wardas, 2008; Shen et al., 2012; Borroto-Escuela et al., 2020). In fact, A_2A_R agonists inhibit hyperlocomotion and sensorimotor gating deficits induced by DR agonists and/or NMDA receptor channel blockers in rodents (Krügel, 2016). More specifically, converging evidence suggests that heteroreceptor complexes containing AR and DR protomers, especially adenosine A_2A_R-D_2_R heteroreceptor complexes, exert strong inhibitory modulation of dorsal and ventral striato-pallidal GABA neurons (Ferrè et al., 1991; Fuxe et al., 2008; Borroto-Escuela et al., 2018; Borroto-Escuela et al., 2020). Thus, A_2A_R agonists reduce D_2_R recognition and function by acting on the A_2A_-D_2_ heteroreceptor complexes located in the dorsal and ventral striato-pallidal anti-reward GABA pathway. Upon activation of this pathway, the brain circuit involved increases the glutamate drive to the frontal cortex from the medial dorsal thalamic nucleus, and transfer of anti-reward information takes place (Fuxe et al., 2008; Borroto-Escuela et al., 2017; Borroto-Escuela et al., 2018; Borroto-Escuela et al., 2020). Thus, it was suggested more than a decade ago (Fuxe et al., 2008) and recently demonstrated (Borroto-Escuela et al., 2020; Valle-León et al., 2020) that drugs promoting A_2A_R-D_2_R heteromer formation might constitute an alternative strategy for the treatment of schizophrenia. Furthermore, A_2A_R agonists can allow a reduction of the dose of the D_2_R antagonists which should reduce the side effects of classical and atypical antipsychotic drugs. These findings moved A_2A_R agonists into the focus of interest for adenosinergic therapeutic options in the disease.

The adenosine A_1_ receptor (A_1_R), too, has been proposed as a potential antipsychotic drug target (Ossowska et al., 2020). A_1_Rs are coupled to the G_i/o_ family of G-proteins, are abundantly present throughout the central nervous system, and appear to generally exert an inhibitory and neuroprotective ‘tone’ (Chen et al., 2014; Krügel, 2016). Activation of presynaptic A_1_Rs inhibits the release of neurotransmitters (e.g., glutamate, GABA, dopamine, serotonin and acetylcholine) and depresses postsynaptic neuronal signaling by inducing hyperpolarization (Paul et al., 2011). Notably, pre- and post-synaptic A_1_R activation, leading to reduced glutamate and GABA release as well as impaired NMDA receptor and D_1_R function, respectively, plays a major role in the “adenosine hypothesis” of schizophrenia (Fuxe et al., 2008; Krügel, 2016). Thus, as the pathophysiologically significant NMDA receptor hypofunction in the disease can be traced mainly to fast-spiking GABA neurons (Nakazawa and Sapkota, 2020), a reduction of A_1_R signaling should benefit critical neuronal circuits and consequently have positive effects on schizophrenia symptoms. In line with this view, A_2A_R agonists might exert part of their antipsychotic action by activating the A_2A_R protomer in a prejunctional A_1_-A_2A_ receptor complex. Through this antagonistic receptor-receptor interaction, A_2A_R agonists could lower the affinity of the A_1_R protomer and thus the inhibitory action of the A_1_R protomer on glutamate release (Ciruela et al., 2006; Franco et al., 2008; Borroto-Escuela et al., 2020). Antagonists of A_1_R receptors have indeed been shown to reduce memory impairment in experimental animals (Boison et al., 2012).

On the other hand, since activation of A_1_Rs on dopaminergic nerve terminals inhibits dopamine release (Paul et al., 2011; Zhang and Sulzer, 2012), A_1_R agonists, too, may counteract schizophrenia symptoms. In fact, preclinical findings have indicated that stimulation of A_1_Rs may have antipsychotic effects, although cognitive dysfunctions must be expected to be associated with the treatment (Ossowska et al., 2020). Specifically, recent studies demonstrated that the selective A_1_R agonist 5-Chloro-5′-deoxy-N6-(±)-(endo-norborn-2-yl)adenosine (5′-Cl-5′-deoxy-ENBA) reduces the hyperlocomotion caused by amphetamine or the non-competitive NMDA receptor antagonist dizolcipine (MK-801; Eyjolfsson et al., 2006; Ossowska et al., 2020). Inhibition of amphetamine- and MK-801-mediated hyperlocomotion may also be caused by allosteric interaction of D_1_R signaling in the A_1_R-D_1_R heteroreceptor complex, which is located in striato-nigral and striato-entopeduncular GABA neurons as well as in D_1_R-rich GABA neurons in the nucleus accumbens (Rimondini et al., 1997; Fuxe et al., 2007; Fuxe et al., 2008; Fuxe et al., 2020; Franco et al., 2008; Pérez-de-la-Mora et al., 2020).

### Kynurenic Acid and Schizophrenia

KYNA, an astrocyte-derived neuromodulator, has been repeatedly linked to the cognitive deficits that are observed in individuals with schizophrenia. KYNA is a metabolite of the kynurenine pathway (KP), which accounts for more than 90% of the degradation of the essential amino acid tryptophan (Cervenka et al., 2017). Through a series of enzymatic steps, the evolutionarily preserved KP generates not only KYNA but also a considerable number of other biologically active compounds, several of which play increasingly appreciated roles in brain physiology and pathology (Schwarcz et al., 2012). KYNA is produced directly from the pivotal KP metabolite kynurenine, either by oxidation (Ramos-Chávez et al., 2018) or by irreversible transamination by kynurenine aminotransferases (KATs; Guidetti et al., 2007). These enzymes are preferentially localized in astrocytes, which promptly release newly formed KYNA into the extracellular compartment (Turski et al., 1989; Guidetti et al., 2007). Though other molecular targets may be of relevance as well, the neurobiological effects of endogenous KYNA are mediated primarily through its actions as an antagonist of both the NMDA and the α7nAChR function, *i.e.* two receptors that are critically involved in cognitive processes (Moroni et al., 2012; Stone et al., 2013; Phenis et al., 2020). Consequently, as shown consistently in experimental animals, elevated brain KYNA levels are associated with a number of cognitive deficits, such as impairments in contextual learning and memory and abnormal visuospatial working memory (Schwarcz et al., 2012; Muneer, 2020). These effects are likely related to the fact that even relatively small fluctuations in KYNA levels bi-directionally affect the extracellular levels of neurotransmitters that play major roles in cognitive functions, including dopamine, acetylcholine, glutamate and GABA (Wu et al., 2007; Zmarowski et al., 2009; Konradsson-Geuken et al., 2010; Beggiato et al., 2013). Notably, selective pharmacological inhibition of KYNA formation has been shown to have pro-cognitive effects in several established animal models (Kozak et al., 2014; Pocivavsek et al., 2019).

The observation that KYNA concentrations are significantly elevated in cortical brain regions and cerebrospinal fluid of individuals afflicted with schizophrenia (Erhardt et al., 2001; Schwarcz et al., 2001; Nilsson et al., 2005; Sathyasaikumar et al., 2011; Linderholm et al., 2012) raised the possibility that KYNA may be causally involved in the cognitive dysfunctions seen in these patients (cf. reviews by Wonodi and Schwarcz, 2010; Erhardt et al., 2017; Plitman et al., 2017; Muneer, 2020). This hypothesis is compatible with the fact that the expression of KYNA’s key biological targets (i.e., NMDA receptors and α7nAChRs) was found to be reduced in the brain of patients with schizophrenia (Guan et al., 1999; Young and Geyer, 2013; Hu et al., 2015). Together with the insights gained from the pre-clinical studies, these findings suggest that interventions leading to a decrease in brain KYNA may constitute a useful strategy for effecting cognitive improvement in the clinical population.

### Adenosine and Kynurenic Acid Interactions: Are They Relevant for Schizophrenia Treatments?

Although neurobiological properties of adenosine may be linked to KYNA, interactions between the adenosine system and the KP have not been carefully examined so far. However, in an *in vivo* microdialysis study performed in rats, local perfusion of adenosine was shown to rapidly and concentration-dependently raise extracellular KYNA levels in the striatum (Wu et al., 2004). Interestingly, this effect was mimicked by perfusion of the A_1_R agonist *N* ^*6*^-cyclopentyladenosine (CPA), whereas the selective A_2A_R agonist *2*-*p*-(2-carboxyethyl) phenylethylamino-5′-N-ethylcarboxamidoadenosine hydrochloride (CGS-21680) was ineffective. Furthermore, local perfusion of the A_1_R antagonist 8-cyclopentyltheophylline (CPT) attenuated the effect of adenosine on extracellular KYNA levels. As the effect of adenosine on KYNA was not observed in the excitotoxically lesioned, i.e., neuron-depleted, striatum, it appears that neuronal A_1_R activation influences glial KYNA synthesis *indirectly* (Wu et al., 2004).

While A_2A_R activation does not appear to affect KYNA levels in the brain under physiological conditions, it is noteworthy that A_2A_Rs not only interact physically with D_2_Rs (see above) but also with the NMDAR (Agnati et al., 2005; Liu et al., 2006). An A_2A_R agonist may therefore inhibit the activity of the D_2_R protomer both in the A_2A_R-D_2_R heteromer (Borroto-Escuela and Fuxe, 2019) and in a putative A_2A_R-D_2_R-NMDAR heteromer, and thereby indirectly enhance NMDAR activity. By this mechanism, A_2A_R stimulation could counteract and reduce the cognitive dysfunction caused by the elevated brain levels of the endogenous NMDAR antagonist KYNA in pathological situations (e.g., schizophrenia).

Furthermore, based on the postulated action of A_2A_R agonists on prejunctional A_1_-A_2A_ heteroreceptor complexes (Ciruela et al., 2006), it also seems possible that A_2A_R agonists, in addition to inhibition of D_2_R signaling, cause a reduction in KYNA levels by allosteric inhibition of A_1_R signaling. In view of the study of Wu et al. (2004); see above), this mechanism, too, may only operate under pathological conditions.

Taken together, these phenomena may have implications for the proposed use of adenosine receptor agonists in the treatment of schizophrenia (Borroto-Escuela et al., 2020). Thus, the beneficial antipsychotic effects of A_1_R agonists, which are predicted from studies in experimental animals (Boison et al., 2012; Ossowska et al., 2020), may also result in cognitive *deficits* due to a A_1_R-induced increase in KYNA levels. Co-treatment with drugs that are able to reduce brain KYNA levels may therefore ameliorate the untoward side effects of A_1_R agonists. Inhibitors of kynurenine aminotransferase II (KAT II), the principal enzyme responsible for the synthesis of rapidly mobilizable KYNA in the mammalian brain (Guidetti et al., 2007), deserve particular attention in this context (Rossi et al., 2010; Nematollahi et al., 2016; Plitman et al., 2017; Blanco-Ayala et al., 2020). Notably, the beneficial effects of KAT II inhibitors may be further enhanced by A_2A_R agonists and may also improve negative symptoms in schizophrenia patients via allosteric inhibition of D_2_R signaling in A_2A_R-D_2_R heteroreceptor complexes of ventral striatal-pallidal GABA neurons (Borroto-Escuela et al., 2020).

## Conclusion

The considerations outlined here indicate a possible relevance of adenosine and KYNA interactions in the pathophysiology and treatment of schizophrenia, and emphasize the need to investigate this issue in detail in future preclinical studies. Specifically, the effects of combined approaches with adenosine receptor ligands and compounds able to reduce brain KYNA levels (e.g., KAT II inhibitors) have not been assessed experimentally so far. Hypothesis testing in rats that were prenatally exposed to kynurenine, which have deficits resembling several of the cognitive impairments seen in schizophrenia patients (Hahn et al., 2018), may be particularly informative for this purpose. These studies may support the development of new multi-target therapeutic strategies that focus on both the purinergic system, especially in relation to adenosine receptor containing heteroreceptor complexes, and brain KYNA function.
